# Recent Development in Micropropagation Techniques for Rare Plant Species

**DOI:** 10.3390/plants9121733

**Published:** 2020-12-08

**Authors:** Vasiliy A. Chokheli, Pavel A. Dmitriev, Vishnu D. Rajput, Semyon D. Bakulin, Anatoly S. Azarov, Tatiana V. Varduni, Victoria V. Stepanenko, Sarieh Tarigholizadeh, Rupesh Kumar Singh, Krishan K. Verma, Tatiana M. Minkina

**Affiliations:** 1 Soil Science and Land Evaluation Department, Academy of Biology and Biotechnologies, Southern Federal University, 344006 Rostov on Don, Russia; vachokheli@sfedu.ru (V.A.C.); pdmitriev@sfedu.ru (P.A.D.); sembakulin@sfedu.ru (S.D.B.); asazarov@sfedu.ru (A.S.A.); varduny@sfedu.ru (T.V.V.); vstepanenko@sfedu.ru (V.V.S.); minkina@sfedu.ru (T.M.M.); 2 Department of Plant Sciences, Faculty of Natural Sciences, University of Tabriz, Tabriz 5166616471, Iran; saryeh_tarigholizadeh@tabrizu.ac.ir; 3 Center of Chemistry, Vila Real (CQ-VR), UTAD, 5000-801 Vila Real, Portugal; rupesh@utad.pt; 4 Key Laboratory of Sugarcane Biotechnology and Genetic Improvement (Guangxi), Ministry of Agriculture and Rural Affairs/Guangxi Key Laboratory of Sugarcane Genetic Improvement/Sugarcane Research Institute, Guangxi Academy of Agricultural Sciences, Nanning 530007, China; drvermakishan@gmail.com

**Keywords:** biotechnology, red-list plants, micropropagation, in vitro, plant tissue culture, rare plants

## Abstract

The current investigation aimed to present an overview of the conservation of biological diversity of rare and endangered plant species. Methods of biodiversity conservation as well as several overview recommendations for the preservation of various rare species have been considered. An overview of the taxa included in the red book has been presented on the example of the Russian Federation. Global and local codes and classifiers of plant rarity were also presented. Future prospects for the conservation of biological diversity and the creation and development of bioresource collections have been considered.

## 1. Introduction

Nowadays, one of the most pressing issues in biology is the preservation of the genetic diversity of living organisms. Special attention is paid to the conservation of the gene pool of many plant species since the most often the price-forming organisms in nature for breeder-preferred traits, as well as consumer-preferred traits [1,2]. The important preserving gene pool is not only for vital agriculture, but also for rare and endangered plants, which often have medicinal, decorative, forage, and other properties. Also, rare plants are important components of vegetation in a particular region. Their disappearance can lead to the destruction of the essence of the biological flora of plant communities [3].

Various plant species in Europe, Asia, America, and in Russian Federation; particularly in the Rostov region of Russia are currently under threat of extinction and have one or another status of rarity. The reasons for the rarity of some plants in this area are mostly the anthropogenic load in the form of plowing land, grazing, construction, and the low competitiveness of the plant species in phytocenoses. Other influencing factors for the propagation of these rare plant species are low seed germination or vegetative reproduction, relict species, torn areas, harsh climatic conditions, eaten by animals and birds, etc. [2,4,5]. Several methods have been adopted to preserve the plant gene pool, for example, (a) collection of closed and open ground-based botanical gardens and nurseries, (b) creation of reserves, and other specially protected natural areas (SPNA), and (c) modern biotechnological tools to create seed banks, cells, tissues, and pollen storage, and organization of banks of plant genetic material, i.e., micropropagation. The modern biotechnological tools have been evolved as the fastest, and most efficient strategies to preserve the plant’s gene pool [2,6,7].

Today, the in vitro culture method is widely used to solve the problems of preservation and restoration of the gene pool of rare and endangered plant species. In addition, this method is able to provide the material in a larger amount for plant breeding programs at specific sites. The endemic habitats of rare and endangered species are often hard to reach for various specific purposes. It is associated, first, with the formation of callus, suspension, meristematic cultures, cultures of ovules, anthers and pollen, cryosurgery of tissues; secondly, the development of technologies for reproduction with the prospect of their further dedifferentiation and redifferentiation. Creating plant in vitro collections can be considered as a protection form of plants of natural flora, and an effective method of their ex situ biodiversity preservation, which is part of the overall strategy for plant protection [6,7,8,9].

## 2. Red-List Plant Species

Currently, 533 plant species are recognized as rare and endangered species in the Russian Federation [10]. On the territory of the Russian Federation, there are more than 85 botanical gardens, and other introduction centers that are the basis for preserving ex situ plant biodiversity. Their main objective is to study and preserve the genetic resources of natural flora [2,11,12,13]. This activity is based on several policy documents at various levels adopted in recent years and titled “Convention on biological diversity” [14,15], “Global strategy for plant conservation” [16], “International program of botanical gardens for plant protection” [17] and “Strategy of botanical gardens of Russia for the conservation of plant biodiversity” [18]. The difficulties of preserving the diversity of the plants in the context of sustainable use of biological resources and the development of biotechnological tools were first considered in the International Convention on biological diversity [19].

Federal and regional legislation establishes the publication of red lists at least once every 10 years. According to the International Union for Conservation of Nature’s Red List of “Threatened Species” (IUCN Red List), there are 43,899 rare and endangered species of fungi and plants worldwide. This list contains information about endangered plant and fungal species from nine phylum, 20 classes, and 579 families. The IUCN Red List website is dynamic and is updated annually with information. To date, the Russian Federation has identified 514 species of wild vascular plants, 61 species of mossy, more than 30 species of fungi, and 42 species of lichenized fungi, or lichens [20]. Whereas in the Rostov region, 1982 species of wild vascular plants, 158 species of mossy, more than 1150 species of fungi, and 192 species of lichenized fungi, or lichens has identified in the Red List of the Rostov region [3]. The latest edition of the “Red List of the Rostov region Vol. 2 “Plants and fungi” [3] listed 273 species of plants and fungi belonging to nine departments, 13 classes, 69 orders, and 99 families.

International Union for Conservation of Nature and Natural Resources (IUCN) identifies the following threat levels: extinct—EX, extinct in the wild—EW, critically endangered—CR, endangered—EN, vulnerable—VU, near to the threatened—NT, least concern—LC, data deficient—DD, not evaluated—NE. According to the International Union for Conservation of Nature’s Red with the help of biotechnological methods, it is necessary to preserve rare plant species that do not reproduce well (for example, low seed reproduction). This is especially true for plants that are important in various types of human activities (Table 1).

For example, several micropropagation protocols have been demonstrated for two rare plant species: *Artemisia hololeuca* Bieb. ex Bess and *Hyssopus angustifolius* Bieb., by the laboratory of cell and genomic technologies of plants in the Botanical garden of the Southern Federal University (SFedU), Rostov-on-Don, Russia.

The is a plant that In the Rostov region *A. hololeuca* Bieb. ex Bess. (*Asteraceae* Bercht. and J. Presl) is under threat of extinction [3,50,51]. The plant is a pillow-shaped shrub, 15–35 cm tall with a strong taproot. Vegetative shoots are numerous, densely pubescent, with numerous leaves, and leaves are tough, twice pinnately dissected. Generative shoots are in a smaller number and leafy. Baskets are in loose panicles, drooping, and multiflowered. Flowers are five-membered, adhesion olepest, with a naked Corolla, yellowish. Achenes are obovate, brownish brown, thinly striated [52]. *Artemisia* in the above ground part contains carotene, alkaloids, essential oil, flavonoids, coumarins; in the roots—traces of alkaloids [53]. For *Artemisia*, there are several studies on introduction [54], population studies [55], and features of seed morphology has been reported [56]. The reasons for the rarity of this species are the extremely small number of localities in the Rostov region, the relict nature of the species (Miocene relict endemic), low competitiveness, and habitat destruction, low seed productivity and amounts to 9.3–12.2% per plant [47].

To preserve the genetic diversity, germplasm is the possibility of further repatriation and creation of a collection of rare and endangered plant species of the Rostov region in vitro. The *A. hololeuca* is a promising plant species for which microclonal propagation technology needs to be developed. The importance of preserving various rare and endangered plant species used by humans has been discussed in many reports. For example, Grigoriadou et al. [8] evaluated in vitro propagation protocols for 22 native medicinal and aromatic plants in Greece, which were assigned conservation priority, and Coelho et al. [7] discussed various cryopreservation methods and assess the cryogenic effects for endemic plant species.

The *H. angustifolius* Bieb. is another rare plant species of the Rostov region from the family *Lamiaceae* Martinov [3]. This is a semishrub with numerous 30–40 cm-high stems, narrowly linear leaves, long inflorescences of two to _six_ flowers in the axils of leaf, tubular-conical calyx with prominent veins, purple-blue corolla, and nut type fruit [57]. The reasons for the rarity of *H. angustifolius* on the territory of the Rostov region include narrow ecological amplitude, low competitiveness, anthropogenic violations of habitats and collection as a valuable medicinal plant [3,50,51]. Many researchers distinguish *H. angustifolius* as a variation of *H. officinalis*, rather than as a separate species [58,59]. The *H. officinalis* and its related species are valuable medicinal plants [60]. Extracts of *H. officinalis* parts have antifungal properties [60], spasmolytic [61], antibacterial [62], tonic the nervous system [63] and antioxidants [64]. Extracts of parts of *H. angustifolius* have antihemolytic properties [65,66] and antioxidant potential [66]. The *H. angustifolius* plants contain many vitamins and macronutrients as well [67]. Preparations based on *H. officinalis* extracts help well with diseases of the respiratory system: cough, bronchitis, and bronchial asthma [68]. There are studies indicating the ability of certain chemical components of *H. officinalis* extracts to inhibit the human immunodeficiency virus (HIV) [69].

## 3. Established Regeneration Techniques for Some of the Rare Plants

The study of biodiversity, the identification of new and assessment of reserves of used types of resources are of theoretical, scientific, and practical significance are currently relevant. The main strategies for the regeneration and conservation of endemic and rare plants are ex situ, in situ, and modern in vitro technologies.

In situ regeneration occurs in natural ecosystems with the creation of specially protected natural territories: nature reserves, national parks, natural monuments etc.

Ex situ regeneration occurs outside of the natural environment such as the collections of botanical gardens, gene banks. These two approaches are fundamentally different: during ex situ conservation, the taxon of interest is removed from the natural environment and cultivated under artificially created conditions, whereas in situ conservation involves determining the habitat and monitoring the plant in situ [70]. The major challenges in organizing protected areas and establishing “living” collections of rare and vulnerable species are the creation, monitoring, and protection of habitats that cover large areas, as well as damage to plants by pests and pathogens [12,71]. It is worth mentioning that the protection of biological diversity in situ is the most preferable, but not always suitable for the conservation of individual species. Therefore, ex situ gene pool conservation strategies are becoming more and more popular. They are based on creating collections of rare and endangered plants with the creation of seed banks and field gene banks [6,70,72].

In addition to traditional methods of ex situ preservation, the use of in vitro culture of isolated tissues and organs is becoming more common. The most effective methods of clonal micropropagation have been optimized for more than 150 species, 1150 varieties, and selected forms belonging to 59 families. The development of effective methods of plant reproduction is the basis for preserving the gene pool. Optimal explants (apical meristem with leaf primordia) were determined for the stable reproduction of plants. Scientific bases of formation and methodological aspects of conservation of rare and valuable plant species in in vitro genetic banks are being developed. Most of the collections of angiosperms are stored in conditions of slow growth (3–7 °C), and the factors affecting the duration of preservation in in vitro have been determined. When creating genetic banks, special attention is paid to the representativeness and preservation of the genetic stability of plant species. One of the most attractive advantages of in vitro conservation is the possibility of obtaining sterile cultures of species (rare, endemic) without removing them from their natural habitat, which helps to prevent the destruction of phytocenoses [71,73,74].

Currently, there are three ways to preserve plants in vitro: storage in conditions of active growth, deposition under slow-growth conditions at low temperatures (+2–15 °C) and cryopreservation in liquid nitrogen (−196 °C). Storing plants in conditions of active growth allows conservationists to successfully preserve, reproduce, and reintroduce for which the use of seeds is difficult due to their low germination and/or requirements for growing conditions. This method is widely used for in vitro conservation of both dicotyledonous and monocotyledonous plants. Besides, for the conservation of rare and endangered species, it is preferable to use cultures of apical shoots and axillary buds to get healthy materials. Activation of meristems existing in the plant also obtains genetically identical offspring [13,75,76].

The most common method of storing plants in vitro is storage in conditions of slow growth, characterized by a decrease in the vegetative activity of the stored material. The main advantage of this approach is the possibility of long-term deposition of plants with lower storage costs, which is provided by increasing the intervals between subcultures [77,78]. Usually in a nutrient medium, plants are subcultured every four to five weeks, but, as mentioned by Molkanova et al. [79] storage of regenerated plants from the Liliaceae family at low temperatures (+3–7 °C), low light on a medium of one half Murashige and Skoog (MS) medium supplemented with 20.0 g/L sucrose and 5.0–7.0 mg/L abscisic acid, can increase the duration of subculturing up to 24 months.

The cultivation of *A. hololeuca* in vitro has still not been investigated. There are known technologies for cultivating some other species of the genus *Artemisia*. For example, *A. vulgaris* demonstrates high values of the multiplication coefficient on MS medium with the addition of 1.0 mg/L 6-(γ,γ-Dimethylallylamino) purine (2-iP). Rhizogenesis of this species is successfully stimulated on the same medium with the addition of indolyl acetic acid (IAA) at a concentration of 0.2 mg/L, providing data on the positive effect of vitamins in concentrations of B5 on the proliferation of *Artemisia* shoots [80]. Furthermore, for *A. vulgaris*, it is recommended to add 1.0 mg/L 6-Benzylaminopurine (BAP) together with 0.5 mg/L kinetin in order to achieve better multiplication on the MS medium. For rhizogenesis, IAA at a concentration of 1.5 mg/L is recommended (data are not given). For *A. annua*, the best medium for multiplication of shoots is MS with the addition of 1.0 mg/L of BAP, and, for rhizogenesis, it is MS with the addition of 5.0 mg/L of Indole-3-butyric acid (IBA) [81]. *A. nilagirica* var. *nilagirica* requires the combined addition of BAP and 2-iP at concentrations of 0.2 mg/L for a high degree of regeneration of shoots from the callus. Rhizogenesis of this species was noticeably observed on MS with the addition of IBA at a concentration of 0.2 mg/L [82]. To obtain the callus of the genus *Artemisia* plants, it was recommended to use Thidiazuron (TDZ) in various concentrations [83]. Sujatha and Ranjitha [69], in their study on *A. vulgaris*, provided data on the positive effect of vitamins in concentrations of B5 on the proliferation of *Artemisia* shoots. The use of surfactants such as Dettol or Tween-20 was indicated in the literature as sterilizing agents during the first stages of sterilization of *Artemisia* explants. To sterilize deeper layers of explants, it was recommended to use 0.1% HgCl_2_ solution and 95% C_2_H_5_OH solution (1:4).

There are some studies on the development of microclonal reproduction technologies for *H. officinalis.* Stem tissue has been labeled as explants in the literature [62]. For bioreactor culture it was possible to use roots as an explant [60]. Among the sterilizing agents used for *H. officinalis* were Tween 20, 2% NaOCl [60], 0.04% HgCl2, 0.1% Tween 20 [62].

Quorin and LePoivre (QL) media with addition of 0.2 mg/L or 0.5 mg/L benzimidazole and 0.5 mg/L PP-40 was suggested as nutrient media for multiplication of *H. officinalis* shoots [62]. For successful in vitro rooting, it was recommended to use the nutrient media supplemented with QL (Quorin and LePoivre), Gamborg (B5), MS (Murashige and Skoog) as whole and with a half concentration of macronutrients [60]. As hormonal regulators of root growth, IAA at a concentration of 0.1 mg/L together with benzimidazole at a similar concentration was recommended [62].

The described approaches do not solve the main problem associated with the high cost of technology, because of the need for periodic subculturing of plants. It is possible to completely abandon subcultures, only if the mitotic activity and metabolic processes are completely stopped. Therefore, as a result, the growth of preserved plants is stopped, which is achieved by cryopreservation methods. Long-term storage of meristems can be achieved by several cryopreservation methods including slow freezing, vitrification (rapid freezing), and encapsulation-dehydration. The first method is based on slow cooling (usually to −40 °C), the use of cryoprotectors, and rapid immersion in liquid nitrogen followed by storage and thawing [84]. The second method, vitrification, involves dewatering samples before rapid cooling by exposure to a highly concentrated cryoprotective solution or air drying, which prevents further formation of intracellular ice crystals [85].

The method of dehydration encapsulation is actively used to create artificial seeds: explants are encapsulated in alginate beads, growing them in a liquid medium, partially dried (airflow, silica gel), and then quickly immersed in liquid nitrogen [74]. Methodically, the regeneration of endangered plant species and endemics directly includes micropropagation from apical meristems and direct somatic embryogenesis to produce artificial seeds. This, combined with the methods of depositing and cryopreservation, allows to create both banks of germplasm of rare and endangered species, and to reintroduce them into the environment.

### 3.1. Examples of Micropropagation Protocols Used for Some of the Rare Plant Species

As explants for *A. hololeuca* and *H. angustifolius*, seeds obtained from the nursery of rare and endangered plant species of the Rostov region of the SFU botanical garden were used. For both species, seeds were sterilized with several substances and solutions in different sequences and with different exposure times. For primary surface sterilization, running water with the addition of a drop of Tween-20 was used. The HgCl_2_ solutions of 0.1%, C_2_H_5_OH 96%, a mixture of 70% C_2_H_5_OH + 3% H_2_O_2_ solution (1:1) were used for further cleaning of explants from infection. Before the inoculation, the seeds were washed in distilled water.

Seeds were sprouted on MS medium [86] without addition of phytohormones. The pH level of the nutrient medium was normalized by using 1 M KOH solution to level 6.3 before autoclaving. The pH level is deliberately overestimated, because after autoclaving, the medium is acidified [87]. Autoclave was performed for 30 min at 121 °C and 1.5 atmosphere in devise MLS-3751L (Sanyo). The plants were cultivated under a 16 h photoperiod and at 23 °C.

#### 3.1.1. Sterilization of *Artemisia hololeuca*

The best method of sterilization was the use of C_2_H_5_OH + H_2_O_2_ followed by washing in distilled water, 100% of the seeds were sterile. It has been observed that the surface of the seeds becomes quickly slimy when liquid gets on it. Most likely, the mucus form when using Tween-20 prevents deep sterilization of seeds. It can be concluded that using a mixture of peroxide and ethanol at the first stage of sterilization can effectively and quickly get rid of the unwanted infection. The 10 min exposure time of the seeds to mercury chloride (HgCl_2_) showed a more satisfactory sterilization result compared to the shorter five minutes exposure time. The sequence of use of the sterilizer was not important for a similar exposure time.

#### 3.1.2. Multiplication of *Artemisia hololeuca*

Of the tested variants of nutrient media, the highest multiplication coefficient of shoots (17.0 ± 1.0) was recorded on MS medium with a twice-reduced concentration of macronutrients and the joint addition of 0.5 mg/L BAP and 0.1 mg/L IBA. This coefficient value significantly exceeds the parameter values for other variants of nutrient media (Table 2). Presumably, the main influence on the multiplication of shoots here is the combined use of cytokinin and auxin, which often has a positive effect on the degree of multiplication of shoots [88,89]. To stimulate the multiplication of *A. hololeuca* shoots, BAP at concentrations of 1 and 2 mg/L and a IBA of 0.1 mg/L were added to the MS medium. In one of the experiment variants, vitamins were used according to the prescription of the B5 medium [90]. The experiments were also performed to reduce the concentration of macronutrients in the MS medium by two to four times.

#### 3.1.3. Rhizogenesis of *Artemisia hololeuca*

The MS medium both without the addition of phytohormones and with the introduction of 2 mg/L IBA showed similar values of the rooting percentage—20% (Table 3). Most likely the concentration of endogenous auxins *A. hololeuca* is high enough that the introduction of additional auxins from the outside inhibits the formation of roots. Thus, by direct organogenesis the technology of micropropagation for *A. hololeuca* was developed. Acclimatization after in vitro cultivation has not yet been investigated. The high efficiency of using a mixture of alcohol and hydrogen peroxide at the initial stages of seed sterilization as explants was revealed. The mucus formation on the seeds when their shell is watered can complicate the process of sterilization of this type of explants. The combined use of cytokinin and auxin in the nutrient medium increases the proliferation rate of *A. hololeuca* shoots. The MS medium without adding additional auxins is optimal for in vitro rhizogenesis of this plant species.

#### 3.1.4. Sterilization of *Hyssopus angustifolius*

The best method of sterilization was the use of C_2_H_5_OH + H_2_O_2_ followed by washing in distilled water, after which 96% of the seeds were sterile. The use of mercury chloride also demonstrated a high degree of sterility of 95%; however, its use was disastrous for the vast majority of *H. angustifolius* seeds because only 5% of sterile seeds managed to germinate. Using 20% NaOCl solution and 96% ethanol was not as successful as previous sterilizers.

#### 3.1.5. Multiplication of *Hyssopus angustifolius*

The QL culture medium with 0.5 mg/L BAP shows best stimulation for *H. angustifolius* in vitro shoots multiplication. Using this nutrient medium, the multiplication coefficient statistically significantly exceeds the values of this indicator for other used nutrient media (Table 4). Thus, the results of our experiment, on the one hand, confirm the information from the literature about the success of using theQL (Quorin and LePoivre) medium for *H. angustifolius* multiplication, on the other hand, show new data on the effectiveness of using BAP instead of benzimidazole and PP-40 [62]. The MS and QL [91] media were used to stimulate the multiplication of *H. angustifolius* shoots without adding phytohormones and with the addition of 0.2 mg/L and 0.5 mg/l BAP. Auxins in various concentrations were used for the formation of roots by plants of *A. hololeuca*: IBA (Indole-3-butyric acid) (2 mg/L, 5 mg/L), IAA (indolyl acetic acid) (2 mg/L), NAA (1-naphthaleneacetic acid) (0.5 mg/L, 0.025 mg/L) and combined application of NAA (1-naphthaleneacetic acid), 0.5 mg/L and BAP (6-Benzylaminopurine) at a concentration of 0.05 mg/L.

#### 3.1.6. Rhyzogenesis of *Hyssopus angustifolius*

Of the 12 tested variants of nutrient media, the highest percentage of rooting in vitro were found on the half MS and MS media—the percentage of rooting in both variants reached 40% (Table 5). At the same time, our data contradict the results of other studies, where WPM (woody plant medium) and QL (Quorin and LePoivre) with the addition of NAA (1-naphthaleneacetic acid) at a concentration of 0.1 mg/L are indicated as the most effective variants of nutrient media for rooting in vitro *H. angustifolius* [59,60]. This may be due to the fact that some of researchers distinguish *H. angustifolius* as a variation of *H. officinalis*, rather than as a separate species, and most of the nutrient media are presented for *H. officinalis* [58]. To activate in vitro Rhizogenesis, *H. angustifolius* plants were transferred to MS (Murashige and Skoog), QL (Quorin and LePoivre), and WPM (woody plant medium) media [92] and they were whole and had a reduced concentration of macroelements. The nutrient media were quenched without the addition of auxins, and with the addition of NAA (1-naphthaleneacetic acid) at a concentration of 1 mg/L. With the statistical analysis used, we determined such indicators as the multiplication coefficient and the percentage of rooting. The values of the proliferation coefficient were compared with each other using Student’s *t*-test. Standard statistical errors were determined for each value.

Thus, we developed a micropropagation technology for *H. angustifolius*. The high efficiency of using a mixture of alcohol and hydrogen peroxide at the initial stages of seed sterilization as explants was revealed. The harmful effect of 0.2% mercury chloride solution on *H. angustifolius* seeds was revealed. The positive effect of BAP at a concentration of 0.5 mg/L on the multiplication of *H. angustifolius* shoots was determined. The MS whole media with a half concentration of macronutrients are quite effective for stimulating the formation of *H. angustifolius* in vitro roots.

## 4. Emerging and Updated Micropropagation Techniques for Rare Plant Species

New methods in emerging and updated micropropagation of rare and endangered species of plants, standard technologies for selecting cultivation conditions are most often discussed. For rare plant species that have not been previously regenerated in vitro, it is very important to optimize the most effective combinations of macro- and microelements, vitamins, amino acids, growth regulators, as well as, sometimes, antibiotics and substances that bind phenolic compounds. It is necessary to take into account the chemical composition of the soil of plant communities where a rare plant grows, which is necessary for introduction into culture [93].

However, today, there are some new techniques and technologies that are useful for in vitro cultivation and which are not only species-specific but also for use with many species of threatened plants that are close in systematic or ecological terms. Recently a robust regeneration method for a crop wild legume and rare plant species “*Cicer microphyllum*” has been demonstrated to conserve the germplasm and to ensure the availability of germplasm for breeding programs [94]. This wild legume has been proved as a natural repository of valuable traits for crop improvement programs [95,96,97,98]. However, this species has endemic habitats in cold deserts of Himalayan mountain regions and diminishing very fast due to overgrazing by animals, and seeds were being eaten by birds. The method optimized for this species could help for large-scale propagation of disease-free plants in a containment facility for breeders to use in a crop improvement program, and may be useful for other rare wild legume species.

Some endangered plants are grown in bioreactor systems. Many studies indicate that growing plant tissues and organs in liquid and semiliquid media in a bioreactor is faster and more efficient than using solid media due to maximum supply of nutrients and hormones to explants, better contact with medium and aeration system for maximum growth for scaling up purposes [99]. Studies using bioreactor systems have been performed for many plant families with a large number of threatened species: *Orchidaceae* Juss. [100], *Araceae* Juss. [101], *Plantaginaceae* Juss. [99], *Rosaceae* Juss. [102], *Asteraceae* Bercht. and J. Presl [103], *Arecaceae* Bercht. and J. Presl, nom. cons. [104], *Moraceae* Gaudich. [105]. Due to their technological properties, bioreactors contribute not only to a fast and high-quality micropropagation process but also to the ability to quickly obtain substances useful for medicine from endangered plant species, especially from roots [100].

However, not all plant species are easily regenerated by in vitro culture. This is especially true for many rare plant species. Cells, tissues, and organs of some rare plant species are difficult to cultivate at one or more stages of micropropagation. These plants are called “recalcitrant” [106]. For “recalcitrant” plants, it is necessary to search for suitable explants, nutrient media, and adaptation methods, which is difficult with tree species, aquatic plants and plants of the family *Orchidaceae* Juss.

When cultivating aquatic plants, biotechnology face a high degree of contamination of plant material, as well as low seed germination [107]. To avoid in vitro contamination of aquatic plants, new types of explants must be sought. A striking example is the representatives of the family *Nymphaeaceae* Salisb family. The species *Nymphaea* ‘Daubeniana’ has special epiphyllous shoots that can be used for micropropagation [108]. However, this is the only type of water lilies that has this feature. For many other water lilies, it is recommended to use unfertilized bud ovaries as explants, or seeds, if any [107]. In the case of orchids, difficulties arise when using seed material devoid of endosperm [109], as well as when adapting them ex situ, since many orchids are highly specialized species adapted to specific habitats. Acclimatization of many orchids, such as the ghost orchid (*Dendrophylax lindenii*), is a multi-stage and long-term process [107].

It is important to note the problem of the symbiosis of orchids with mycorrhizal fungi. Due to the almost complete lack of nutrients in the seeds of orchids, these plants are forced to enter into a symbiotic relationship with some mycorrhizal fungi. Without mycorrhizal fungi, even with micropropagation, the cultivation of orchids can become almost impossible. Therefore, in the case of orchids, introducing fungal cultures into in vitro cultures specific to each particular type of orchid is actively practiced. This technology is particularly important for the acclimatization of orchids to indoor and outdoor conditions [110].

The basis for rapid growth and obtaining certain organs and tissues at a particular stage of micropropagation is the use of various growth regulators. Among the many commonly used phytohormones, rarer substances sometimes appear in vitro. One of these is triacontanol, which stimulates the formation of chlorophyll and increases the intensity of photosynthesis. The effect of the presented phytohormone is described on such plants as sweet wormwood (*A. annua*) [111], apple (*Apple domestica*) [112], lemongrass (*Cymbopogon flexuosus*) [113], noble dendrobium (*Dendrobium nobile*), etc. [114].

Protoplast culture is sometimes used to preserve and study the genetic component of rare and endangered plants. This provides great convenience when extracting the genome or transcriptome [115]. Recently, some narrowly focused techniques used for micropropagation of plants have been developed. These include in vitro micrografting and facilitates the cultivation of many woody plants [116]. An interesting and important problem is the hyper hydracity of explants. It can interfere with the growth and rooting of regenerating plants. To get rid of waterlogging, various polishing agents are used that differ from the usual hangar [117].

A relatively new technique in the micropropagation of both rare and many other plants is biotization of endophytic microorganisms. This technology is designed to stimulate growth, development, reduce stress, and increase plant immunity in vitro by introducing bacteria and fungi into cultures [118]. New studies have revealed a positive effect of many microorganisms on the growth of the vegetative part of plants, seed maturation, resistance to pathogens, callus growth, and increased tolerance to low temperature [119].

## 5. Future Perspectives

**Figure 1:**
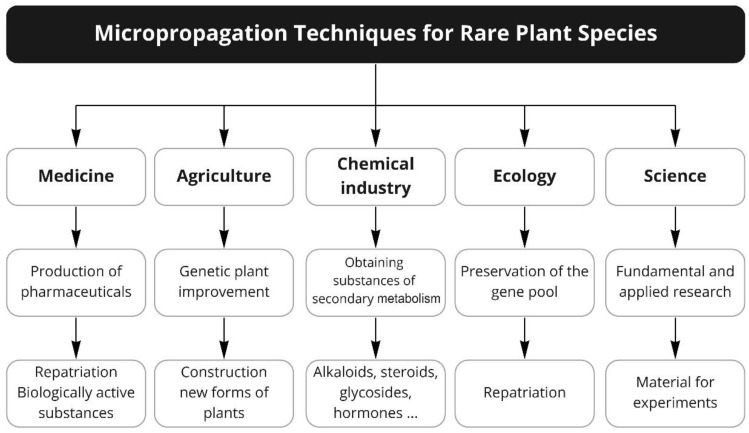
Application of micropropagation techniques for rare plant species.

Conservation of diversity is one of the main mechanisms that support the stability of life on earth, while the disappearance of certain plant species may result in an increase in imbalance of natural fitness and lead to a shortage of raw materials that could be used for specific traits in crop improvement programs, medicine, the chemical industry, etc. (Figure 1). Briefly, the technology of micropropagation of rare and endangered plant species can act as a base for:

- conservation of the gene pool of rare plant species (cryopreservation);
- study of the resistance of “red list” plant species to various adverse abiotic and biotic environmental factors;
- repatriation of rare and endangered plant species to plant community to restore disturbed vegetation cover;
- genetic improvement of endangered plant species;
- creating hybrid forms of plants;
- design of cells through the introduction of various cellular organelles;
- synthesis of new unusual compounds;
- obtaining secondary synthesis substances that are valuable for medicine, perfumes, cosmetics, and other industries;
- a storehouse of a variety of gene pool for various breeder preferred and consumers preferred traits; especially in these rapidly changing climatic conditions.

Thus, at present, progress in medicine, agriculture, and the chemical industry are closely linked to the further development of plant cell technologies, and this is one of the most promising tools for preserving rare and endangered plant species and biodiversity in general.

Remote sensing methods offer great prospects for rapid and noninvasive monitoring of rare and endangered plant species. Various types and combinations of sensors are used for this purpose [120,121,122,123]. The most promising is hyperspectral cameras [124], which can receive multiple frames of the same scene in different adjacent ranges of the electromagnetic spectrum. Today, there are many approaches to using hyperspectral survey data to identify the species of agricultural, woody, and other plant species and varieties [125,126].

In our work, we observed prospects for their use for monitoring rare and endangered plant species. For these purposes, it is necessary to create spectral libraries of “red list” plant species. In this case, cell and tissue culture can be used as a base for obtaining experimental samples of “red list” plant species to develop a similar technology that allows remote and short-term monitoring of rare and endangered plant species.

## 6. Conclusions

To preserve rare and endangered plant species, along with classical methods of ex situ conservation, it is necessary to create so-called bioresource collections. Bioresource collections are not only physical stores of biological material (such as in vitro culture, callus cultures, DNA Bank, cryopreservation of samples, seed library, etc.) but also specialized databases containing a complete description of species from the collection of rare and endangered plant species (including botanical descriptions of populations, data on the genetic structure of populations, data on plant genotyping, genetic passports, etc.) posted on the Internet in open access. The data of bioresource collections must be updated with new data and samples of plants. The goals of bioresource collections are diverse: they could be of great scientific interest, and may serve educational purposes for students and researchers worldwide. The creation of such bioresource collections with a database will help the collaboration of research groups in large botanical gardens, which will more effectively preserve the biodiversity of rare and endangered plant species.

## Figures and Tables

**Table 1:** Some of the rare plants in human use.

Species	Family	IUCN Red List Category and Criteria or Status of Rare Plants in Russia	Threats	Use and Trade	Citation
***Isoplexis isabelliana***	Scrophulariaceae	Endangered B2ab(iii); C2a(i)	Housing and urban areas, livestock farming and ranching, invasive non-native/alien species/diseases	Medicine—human and veterinary, food—animal	[21]
***Lavandula maroccana***	Lamiaceae	Vulnerable B2ab(ii,iii,iv)	Tourism and recreation areas, annual and perennial nontimber crops, gathering terrestrial plants, work and other activities, habitat shifting and alteration.	Medicine—human and veterinary, food—human, food—animal	[22]
***Cypripedium montanum***	Orchidaceae	Vulnerable B2ab(ii,iii,iv,v)	Tourism and recreation areas, annual and perennial nontimber crops, wood and pulp plantations, livestock farming and ranching, agricultural and forestry effluents	Medicine—human and veterinary, pets/display animals, horticulture	[23]
***Medicago sativa***	Fabaceae	Least concern	Hybridization/introgression between the crop and wild populations	Medicine—human and veterinary, food—human, food—animal	[24]
***Voanioala gerardii***	Arecaceae	Critically endangered D	Annual and perennial nontimber crops, gathering terrestrial plants, logging and wood harvesting	Pets/display animals, horticulture, food—human	[25]
***Amorphophallus titanum***	Araceae	Endangered A2ac; C2a(i); D	Annual and perennial nontimber crops, gathering terrestrial plants logging and wood harvesting, fire and fire suppression	Fire and fire suppression, medicine—human and veterinary, food—human	[26]
***Oryza officinalis***	Poaceae	Least concern	Commercial and industrial areas, renewable energy, dams and water management/use, storms and flooding	Food—human	[27]
***Santolina oblongifolia***	Asteraceae	Near threatened	Livestock farming and ranching, gathering terrestrial plants, fire and fire suppression, problematic species/disease of unknown origin	Food—animal, medicine—human and veterinary	[28]
***Bufonia multiceps***	Caryophyllaceae	Endangered B1ab(i,ii,iii,v) + 2ab(i,ii,iii,v); C2a(i)	Livestock farming and ranching, gathering terrestrial plants, dams and water management/use, recreational activities, work and other activities, invasive nonnative/alien species/diseases	Food—animal, medicine—human and veterinary	[29]
***Anemone halleri***	Ranunculaceae	Least concern	Gathering terrestrial plants, recreational activities	Medicine—human and veterinary, pets/display animals, horticulture, establishing ex situ production	[30]
***Acer japonicum***	Sapindaceae	1—Species under threat of extinction	Small number of individuals in the population, lack of seed renewal	Ornamental plant.	[31]
***Galanthus caucasicus* (Baker) Grossh.**	Amaryllidaceae	3 d—Rare species with a limited range, part of which is located on the territory of Russia	Suffers from mass harvesting of flowering plants and the digging of bulbs for commercial purposes. Habitat disturbance, deforestation. Low seed productivity. Weak frost resistance.	Decorative, honey-bearing, poisonous, medicinal plant.	[32]
***Sternbergia colhiciflora* Waldst. et Kit.**	Amaryllidaceae	1—Species under threat of extinction	Destruction of natural habitats during economic activities (plowing, construction, etc.). Weak seed renewal. Suffers from collecting and digging bulbs.	Ornamental plant.	[33]
***Kalopanax septemlobus* (Thunb.) Koidz.**	Araliaceae	3 g—A rare subtropical species on the northern border of distribution, with a significant gap from the main range	Felling for valuable wood, forest fires, irregular seed maturation, poor vegetative reproduction, reduced ability to reproduce seeds.	Medicinal, food plant, wood is used for making musical instruments.	[34]
***Arnica alpina* (L.) *Olin***	Asteraceae	2 a—Species that is declining in number as a result of changing living conditions	Low vitality due to weakened seed renewal, narrow ecological amplitude. Industrial development of mountain ranges, increasing recreational loads.	Ornamental plant.	[35]
***Brachanthemum baranovii* (Krasch. et Pol.) Krasch. excl. typo**	Asteraceae	1—Species under threat of extinction	Narrow ecological range of the species, weak seed renewal. Mining and road works, grazing.	Ornamental plant. Endemic to the Altai Mountains.	[36]
***Ostrya carpinifolia Scop.***	Betulaceae	2 a—Species that is declining in number as a result of changing living conditions	Deforestation, fires, and uncontrolled grazing. Strict adherence to carbonate rocks, weak seed renewal, low germination, late entry into fruiting, low competitiveness.	Source of timber.	[37]
***Pueraria lobata* (Willd.) Ohwi**	Fabaceae	3 g—Rare subtropical species on the northern border of distribution, with a significant gap from the main range	Instability to frost. Small and disjointed populations, low seed productivity, economic activity, fires and grazing	Ornamental plant, and soil-strengthening plants.	[38]
***Adlumia asiatica* Ohwi.**	Papaveraceae	2 a—Species that is declining in number as a result of changing living conditions	Difficult seed reproduction. Weak competitive ability of the species. Human economic activity (deforestation, plowing of land), recreation, territory development.	Ornamental plant	[39]
***Nuphar japonica* DC.**	Nymphaeaceae	1—Species under threat of extinction	Natural—limited range, sensitivity to water pollution, changes in the water regime, poor seed productivity. Anthropogenic impact.	Ornamental and medicinal plants.	[40]
***Astragalus dasyanthus* Pall.**	Fabaceae	4—Undefined by status	Seed productivity is low, and seeds are often damaged by pests and diseases	A valuable medicinal, honey-bearing, ornamental plant.	[41]
***Astragalus ponticus* Pall.**	Fabaceae	3 e—Rare species having a limited range, part of which is located on the territory of the Rostov region	Seed productivity is low. The coefficient of semnificatia—13%	Ornamental plant	[42]
***Calophaca wolgarica* (L. f.) DC.**	Fabaceae	2—Declining in number. Taxa with steadily decreasing numbers, which, under the further influence of factors that reduce the number, may in a short time fall into the category of endangered: 2 a—taxa whose number is reduced as a result of changes in the conditions of existence or destruction of habitats	Seed productivity is low, field germination of seeds is 25–60%	Decorative, honey-bearing, fibrous, antierosion plant; promising for introduction to gardening	[43]
***Aldrovanda vesiculosa* L.**	Droseraceae	1 b—Taxa and populations that are at high risk of loss due to extremely low numbers and/or narrow range or extremely limited number of locations.	Seed productivity is low due to the need for high water temperature for normal development (23–30 °C), at 17 °C growth is inhibited	Ornamental, forage, and aquarium.	[44]
***Polygonatum multiflorum* (L.) All.**	Convallariaceae	3 d—Having a significant range, but located in the Rostov region on the border of distribution	Seed productivity is low	Medicinal, ornamental plant, cosmetic, poisonous plant	[45]
***Jurinea cretacea Bunge* (incl. *J. talievii* Klok.)**	Asteraceae	3 b (3). Rare species with a narrow ecological amplitude associated with a specific substrate for production	Seed productivity is low. The coefficient of semnificatia—33.5%	Ornamental plant	[46]
***Erysimum cretaceum* (Rupr.) *Schmalh. (E. ucranicum auct. non J. Gay)***	Brassicaceae	3 c, d—Rare species with a narrow ecological amplitude, associated with a specific substrate for growth and having a limited range, part of which is located on the territory of the Rostov region; Pliocene relic	Seed productivity is low. The coefficient of semnificatia from 10.5 to 46.5%	Ornamental plant, antierosion plant	[47]
***Iris pumila* L.** **[*I. pumila* L. *subsp. taurica* (Llod.) *Rodion. & Shewcz., I. taurica* Llod.]**	*Iridaceae*	2 a—Declining in number. Taxa whose number is reduced as a result of changes in the conditions of existence or destruction of habitats	Seed productivity is low. The coefficient of semnificatia 33.3%	Ornamental plant	[47]
***Hyacinthella pallasiana* (Stev.), *Losinsk.***	*Hyacinthaceae*	3 c, d—Rare species with a narrow ecological amplitude, associated with a specific substrate for growth and having a limited range, part of which is located on the territory of the Rostov region; Pliocene relic	Seed productivity is low. The coefficient of semnificatia 55–60%	Ornamental plant	[48]
***Eremurus spectabilis* Bieb.**	*Asphodelaceae*	2 a—Declining in number. Taxa whose number is reduced as a result of changes in the conditions of existence or destruction of habitats	Seed productivity is low. The coefficient of semnificatia 16.5–60%	Medicinal, ornamental plant,	[49]

**Table 2:** Ratio proliferation of shoots of *Artemisia hololeuca* in a variety of media.

MS	MS + 1 mg/L BAP	MS + Vitamins B5 + Sucrose 3% + 1 mg/L BAP	½ MS	1/4 MS + 2 mg/l BAP	½ MS + 0.5 mg/L BAP + 0.1 mg/L IBA
3.4 ± 0.31	3.8 ± 0.9	8.3 ± 1.6	4.0 ± 0.63	3.4 ± 0.62	17.0 ± 1.0

MS—Murashige and Skoog medium, BAP—6-Benzylaminopurine, IBA—Indole-3-butyric acid.

**Table 3:** Percentage of rooting of *Artemisia hololeuca* in a variety of media.

MS + 2 mg/L IBA	MS + 2 mg/L IAA	MS + 5 g/L IBA	MS + 0.05 mg/L BAP + 0.5 mg/L NAA	MS + 0.025 mg/L NAA	MS
20%	10%	0%	10%	10%	20%

MS—Murashige and Skoog medium, IBA—Indole-3-butyric acid, IAA—indolyl acetic acid, NAA—1-naphthaleneacetic acid.

**Table 4:** Proliferation coefficient of *Hyssopus angustifolius* shoots on different media.

QL	QL + 0.2 mg/L BAP	QL + 0.5 mg/L BAP	MS	MS + 0.2 mg/L BAP	MS + 0.5 mg/L BAP
4.5 ± 0.69	6.5 ± 1.11	7.3 ± 0.75	4.0 ± 0.71	2.2 ± 0.59	1.0 ± 0.80

MS—Murashige and Skoog medium, BAP—6-Benzylaminopurine, QL—Quorin and LePoivre medium.

**Table 5:** The percentage of rooting of *Hyssopus angustifolius* in different media.

Medium	Percentage of Rooting
½ QL	30%
QL	20%
½ QL + 1 mg/L NAA	10%
QL + 1 mg/L NAA	5%
½ MS	40%
MS	40%
½ MS + 1 mg/L NAA	30%
MS + 1 mg/L NAA	20%
½ WPM	20%
WPM	30%
½ WPM + 1 mg/L NAA	30%
WPM + 1 mg/L NAA	20%

QL—Quorin and LePoivre medium, NAA—1-naphthaleneacetic acid, MS—Murashige and Skoog medium, WPM—woody plant medium.
